# Primary gastric actinomycosis: report of a case diagnosed in a gastroscopic biopsy

**DOI:** 10.1186/s12907-015-0002-8

**Published:** 2015-02-26

**Authors:** Khaleel Al-Obaidy, Fatimah Alruwaii, Areej Al Nemer, Raed Alsulaiman, Zainab Alruwaii, Mohamed A Shawarby

**Affiliations:** Pathology Department, College of Medicine, University of Dammam, P.O. Box 1982, Dammam, 31441 Saudi Arabia; Department of Internal Medicine, College of Medicine, University of Dammam, P.O. Box 1982, Dammam, 31441 Saudi Arabia; King Fahd Hospital of the University, University of Dammam, P.O. Box 2208, Al-Khobar, 31952 Saudi Arabia

**Keywords:** Actinomycosis, Gastric, Grocott’s, Gram, PAS

## Abstract

**Background:**

Primary gastric actinomycosis is extremely rare, the appendix and ileocecal region being the most commonly involved sites in abdominopelvic actinomycosis. Herein, we report a case of primary gastric actinomycosis. The diagnosis was made on microscopic evaluation of gastroscopic biopsy specimens. To the best of our knowledge, this is the third case to be reported in the literature, in which the diagnosis was made in a gastroscopic biopsy rather than a resection specimen.

**Case presentation:**

An 87-year-old Saudi male on medication for cardiomyopathy, premature ventricular contractions, renal impairment, hypertension, and dyslipidemia, presented to the emergency department with acute diffuse abdominal pain, abdominal distension, constipation and vomiting for two days, with no history of fever, abdominal surgery or trauma. The patient was admitted to the hospital with an impression of gastric outlet obstruction. Based on radiologic and gastroscopic findings, a non-infectious etiology was suspected, possibly adenocarcinoma or lymphoma. Gastroscopic biopsies showed an actively inflamed, focally ulcerated atrophic fundic mucosa along with fragments of a fibrinopurulent exudate containing brownish, iron negative pigment and abundant filamentous bacteria, morphologically consistent with *Actinomyces*.

**Conclusion:**

Althuogh extremely rare, primary gastric actinomycosis should be considered in the differential diagnosis of radiologic and gastroscopic diffuse gastric wall thickening and submucosal tumor-like or infiltrative lesions, particularly in patients with history of abdominal surgery or trauma, or those receiving extensive medication. A high level of suspicion is required by the pathologist to achieve diagnosis in gastroscopic biopsies. Subtle changes such as the presence of a pigmented inflammatory exudate should alert the pathologist to perform appropriate special stains to reveal the causative organism.

## Background

Actinomycosis is a chronic suppurative granulomatous inflammation caused by anaerobic, filamentous, Gram-positive bacteria of *Actinomyces* species, most often *Actinomyces israelii*. There are three main forms of actinomycosis, namely, cervicofacial (31%-65%), abdominopelvic (20%-36%) and thoracic (15%-30%) [1-4]. In abdominopelvic actinomycosis, the appendix and ileocecal region are the most commonly involved sites (65%) [2,3,5-7]. Primary gastric actinomycosis is extremely rare, with only 23 cases reported to date [5,6,8-20]. Herein, we report a case of primary gastric actinomycosis. The diagnosis was made on microscopic evaluation of gastroscopic biopsy specimens. To the best of our knowledge, this is the third case to be reported in the literature, in which the diagnosis was made in a gastroscopic biopsy rather than a resection specimen [6,8].

## Case presentation

### Clinical and laboratory findings

**Figure 1:**
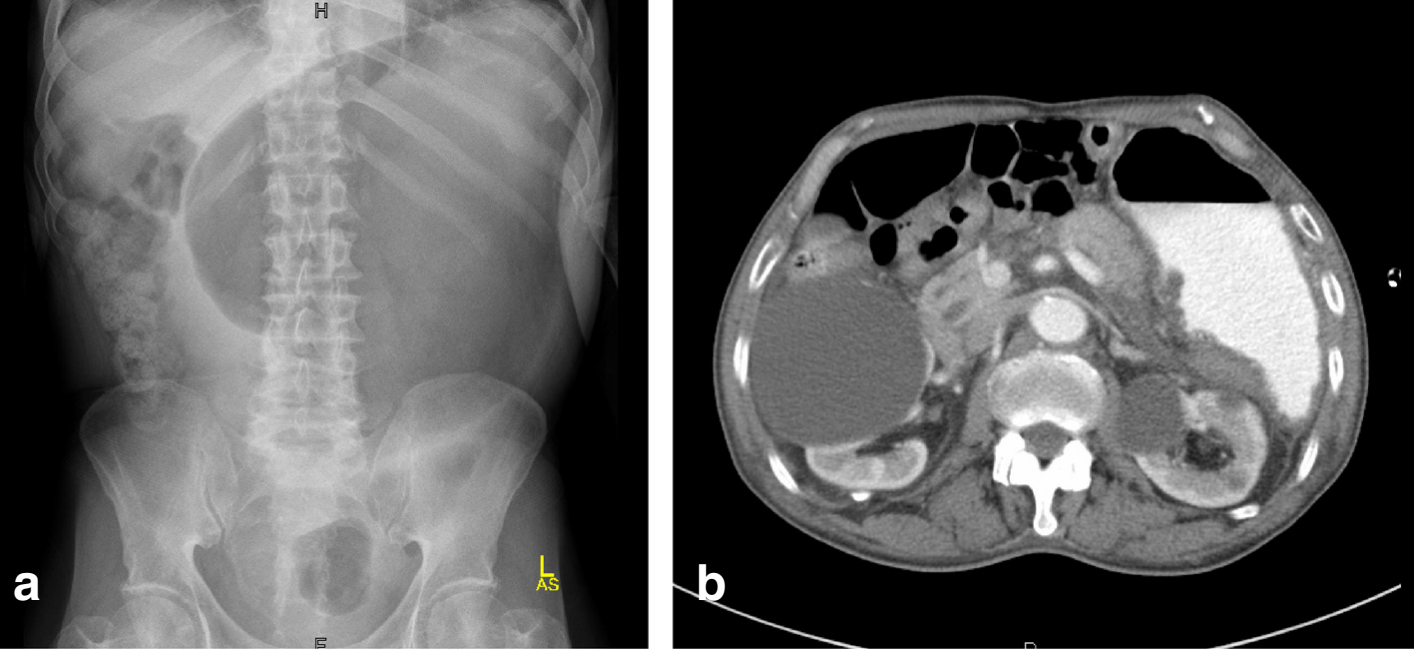
**Radiologic findings. a**. Plain abdominal X-ray showing a markedly dilated stomach **b**. Contrast CT-scan showing a significantly distended stomach with thickened wall and abnormal configuration. Note also air-fluid level.

**Figure 2:**
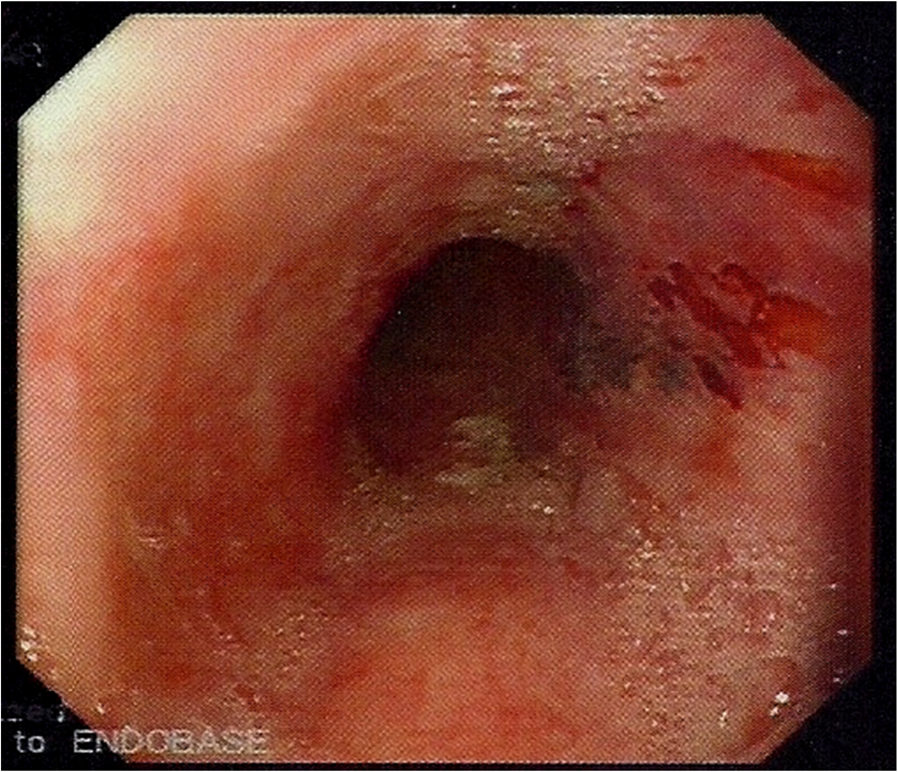
**Gastroscopy.** Deformed stomach, with rotation like appearance. There are inflamed areas with greenish brown material over the surface.

**Figure 3:**
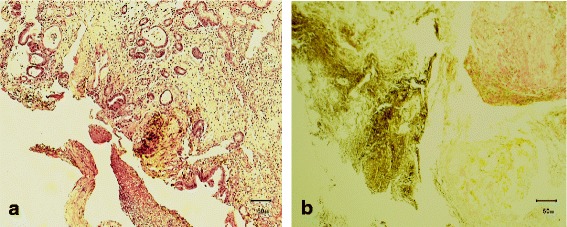
**Gastroscopic biopsies. a**. Inflamed atrophic mucosa with dilated foveolae and pigmented fibrinopurulent inflammatory exudate. H & E x 100 **b**. Perl’s stain showing iron negative brownish pigment. x 400.

**Figure 4:**
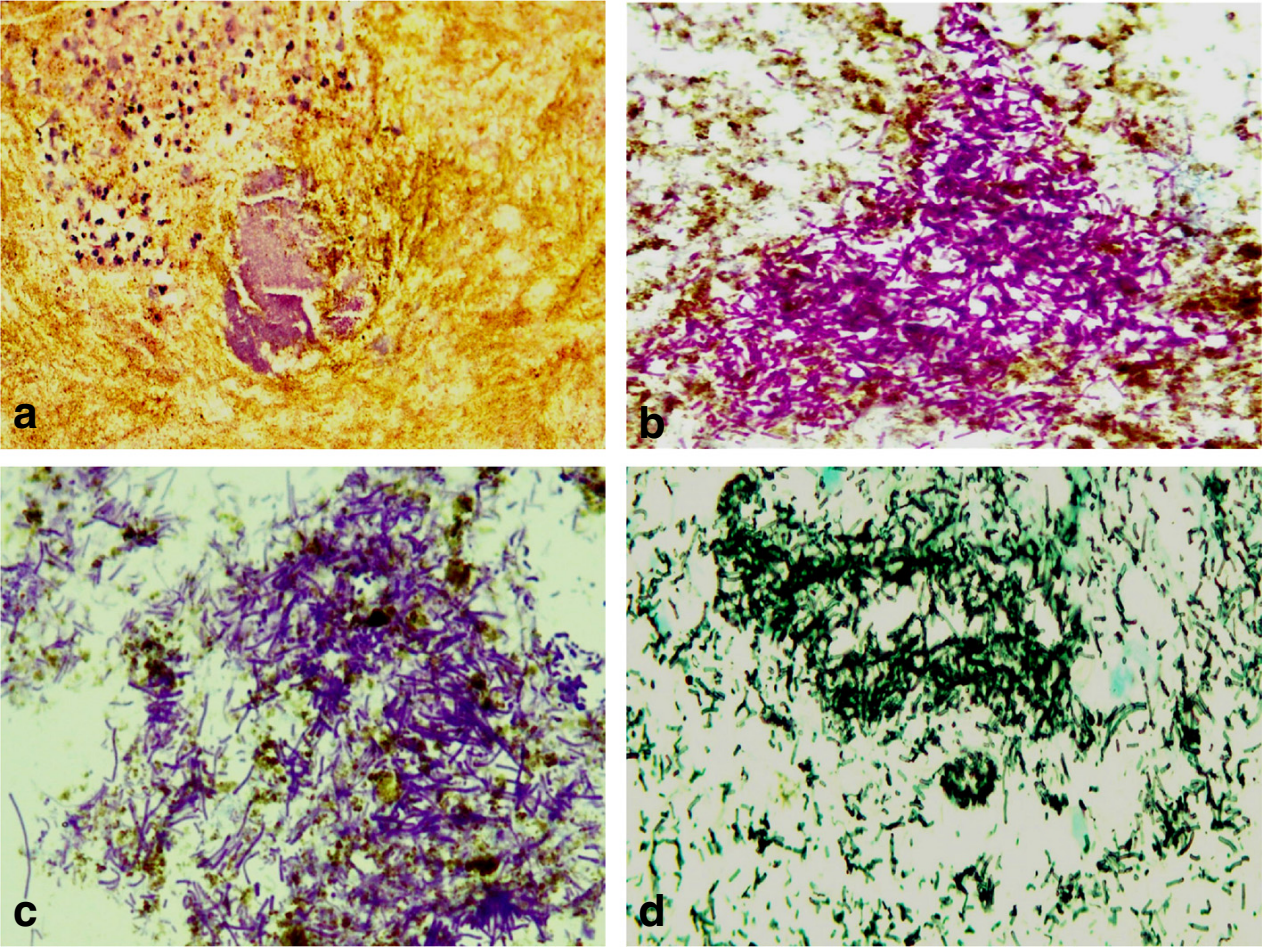
**Filamentous and rod-like bacteria consistent with** ***Actinomyces*** **in gastroscopic biopsies. a**. H & E x 400 **b**. PAS x 1000 **c**. Gram x 1000 **d**. Grocott’s x 1000.

An 87-year-old Saudi male on medication for non-ischemic cardiomyopathy, frequent premature ventricular contractions, renal impairment, hypertension, and dyslipidemia, presented to the emergency department with acute diffuse abdominal pain, abdominal distension, constipation and vomiting of two days duration, with no history of fever, abdominal surgery or trauma. Medications received by the patient for the last four years included, mainly, daily acetyl salicylic acid 81 mg, atorvastatin 40 mg, irbesartan 300 mg and hydrochlorothiazide 25 mg. Abdominal examination revealed stable vital signs along with positive findings of abdominal distension and mild epigastric tenderness. Laboratory investigations showed leucocytosis (16.6 k/μl with 89% segmented cells), mild normocytic normochromic anemia (Hgb 11.5 g/dl, MCV 93.7 fl, MCH 31.7 pg), elevated serum lipase (1123 U/L), amylase (269 U/L), and creatinine (1.4 mg/dl), and low potassium (3.1 mEq/L). Plain abdominal X-ray showed a markedly dilated stomach (Figure 1a). The patient was admitted to the hospital with an impression of gastric outlet obstruction. NGT was inserted & aspiration yielded a large amount of greenish fluid. The patient was then immediately put on empiric antibiotic coverage for 5 days with 2 doses of IV levofloxacin and 3 doses of IV metronidazole administered. Contrast CT-scan, performed to rule out an organic cause for the gastric outlet obstruction, showed a significantly distended stomach with thickened wall and abnormal configuration, and a single air-fluid level (Figure 1b). Two gastroscopies were then performed, 1 week apart, and revealed a deformed stomach with a hard mass infiltrating the greater curvature in the fundic area, covered by necrotic greenish brown material, along with absent peristaltic movement and no apparent organic obstruction to the gastric outlet (Figure 2). Based on the radiologic and gastroscopic findings, a non-infectious etiology was suspected, possibly adenocarcinoma or lymphoma. Biopsies obtained from the edge and the centre of the fundic mass during both gastroscopies were sent for pathological examination. Histologic examination showed an actively inflamed, focally ulcerated, atrophic fundic mucosa with variable, focal eosinophilic infiltration, edema, and variably dilated foveolae with focal regenerative epithelial atypia (Figure 3). There were also fragments of a fibrinopurulent exudate mixed with brownish, iron negative pigment (Perl’s stain) and abundant PAS, Grocott’s, and Gram positive rod-like and filamentous bacteria, morphologically consistent with *Actinomyces* (Figure 4). The organisms were overlooked in the first biopsy. The second biopsy was performed because a diagnosis of malignancy was still in suspicion, despite the negative result of the first biopsy. A revisit to the first biopsy confirmed negativity for malignancy but revealed the presence of organisms identical to those noted in the second biopsy. Culturing of gastric contents following the second gastroscopy, yielded only *Streptococcus viridans* with no *Actinomyces* identified. However, anaerobic culture was not specifically ordered by the clinician. Consequently *Actinomyces*, known to be strictly anaerobic, were not detected. Despite the negative culture, the typical morphology of the organisms in tissue sections confirmed by positive Grocott, PAS and Gram staining was considerd sufficient for diagnosis with no necessity for confirmation by repeat culturing under anaerobic conditions.

The patient was then managed conservatively in the hospital. A third gastroscopic biopsy two weeks later revealed chronic atrophic gastritis with no *Actinomyces* detected, and the patient appeared in a good health status. A plan was set up to start him on the appropriate antibiotic therapy for actinomycosis with follow up gastroscopy after one month. However, the patient chose to continue treatment somewhere else. So he was discharged on his request and never showed up again in our institution.

## Discussion

Actinomycosis in human is most commonly caused by *Actinomyces israelii* [1,3,21-26] which is an endogenous commensal present in the oral and GI-tract flora [9,10,12,22,27]. Actinomycetes typically invade injured mucosa with opportunistic infection occuring if there is a break in the mucosal barrier. Factors that precipitate intra-abdominal actinomycosis include GI surgery, inflammation, and visceral perforation [28,29]. However, in most cases of gastric actinomycosis, it has been impossible to trace the mechanism by which *Actinomyces* had reached the gastric wall [30]. Our patient had no past history of abdominal surgery or trauma. However, he was on prolonged medication for non-ischemic cardiomyopathy, premature ventricular contractions, renal impairment, hypertension, and dyslipidemia. Such extensive medication may have caused physical or functional gastric mucosal damage that facilitated entry of the organisms into the gastric wall. Numerous drugs, acting through various mechanisms, have been associated with gastric mucosal damage [31]. Age related mucosal atrophy may have also contributed to diminished mucosal resistance.

The rarity of gastric involvement by actinomycosis has been attributed to the high lumenal acidity of the stomach. As a result of the low gastric pH, the organisms may be killed or growth is inhibited [9].

The usual presenting clinical manifestations of gastric actinomycosis are low-grade fever, epigastric pain, weight loss, and upper GI bleeding [1,3,10,12,20]. One patient developed symptoms of gastric outlet obstruction [19]. The duration of symptoms ranged from two weeks to several years [3,8,9,11,19]. Our patient presented with acute diffuse abdominal pain, abdominal distension, constipation and vomiting for two days duration, with no history of fever. The clinical impression was that of gastric outlet obstruction.

There is no specific radiological or endoscopic appearance for gastric actinomycosis. CT findings have mostly demonstrated an infiltrative lesion with diffuse gastric wall thickening. The appearance suggested adenocarcinoma or lymphoma of the stomach [2,20,32]. In our case, contrast CT-scan showed a significantly distended stomach with thickened wall and abnormal configuration. Similar to radiologic studies, the endoscopic findings of the disease may simulate a gastric neoplasm and include submucosal tumor-like or infiltrative lesions and, occasionally, mucosal ulceration [13]. A non-infectious etiology was initially suspected in our patient based on radiologic and endoscopic findings, possibly adenocarcinoma or lymphoma, and the gastric outlet obstruction subsequently interpreted as functional due to absence of peristaltic movement consequent to infiltration of the gastric wall by actinomycosis. An associated paralytic ileus due to acute pancreatitis may be an alternative explanation for the obstruction as suggested by elevated serum lipase and amylase levels. Such obstruction may have also contributed to the gastric localization of the actinomycosis, so that the clinical manifestations may be a consequence of acute pancreatitis with secondary gastric overinfection by *Actinomyces*, facilitated by the mucosal damage.

Because of the submucosal localization of the inflammatory process, gastroscopic biopsy specimens usually reveal nonspecific inflammatory changes [3,14,18,19]. In most cases, the diagnosis was made after surgery and histopathological examination of the resected specimen [9,12,19,20,22]. Only two cases have been reported in which the diagnosis of gastric actinomycosis was made on microscopic evaluation of a gastroscopic biopsy specimen [6,8]. In our case, the diagnosis was, likewise, established through histologic examination of gastroscopic biopsies in which abundant PAS, Grocott’s, and Gram positive rod-like and filamentous bacteria, morphologically consistent with *Actinomyces* were identified. The presence of a brownish, iron negative pigment in the fibrinopurulent inflammatory exudate (that was also visible endoscopically) alerted us to the possibility of actinomycosis which was established by appropriate special staining that revealed the microorganisms. It is well known that the main sources of natural pigments are plants and microorganisms, including *Actinomycets* [33].

Culturing is negative in most cases of gastric actinomycosis (>76%) [19,24,25]. In our case, culturing yielded only *Streptococcus viridans*, another endogenous aerobic/anaerobic facultative commensal present in the oral and GI-tract flora [34]. Despite the negative culture, the typical morphology of the organisms in tissue sections confirmed by positive Grocott, PAS and Gram staining was considered sufficient for the diagnosis of *Actinomyces* infection with no necessity for culture confirmation.

Most anaerobic bacteria recovered from clinical infections are found mixed with other anaerobic organisms [35]. Polymicrobial infections are known to be more pathogenic for experimental animals than are those involving single organisms [35]. Whether *Streptococcus viridans*, known to be an organism of low virulence, had contributed to the gastritis in our case remains unclear.

Primary gastric actinomycosis is an indolent infection. If the disease is recognized, the prognosis is good because antibiotic treatment, particularly penecillin is very effective [4,19]. Our patient received 2 doses of IV levofloxacin and 3 doses of IV metronidazole and appeared in a good health status, two weeks after diagnosis.

## Conclusions

Althuogh extremely rare, primary gastric actinomycosis should be considered in the differential diagnosis of radiologic and gastroscopic diffuse gastric wall thickening and submucosal tumor-like or infiltrative lesions, particularly in patients with history of abdominal surgery or trauma or those receiving extensive medication. A high level of suspicion is required by the pathologist to achieve diagnosis in gastroscopic biopsies. Subtle changes such as the presence of a pigmented inflammatory exudate should alert the pathologist to perform appropriate special stains to reveal the causative organism.

## Consent

Written informed consent was obtained from the patient for publication of this case report and any accompanying images. A copy of the written consent is available for review by the Editor of this journal.

## References

[1] Choi MM, Beak JH, Lee JN, Park S, Lee WS (2009). Clinical features of abdominopelvic actinomycosis: report of twenty cases and literature review. Yonsei Med J.

[2] Isik B, Aydin E, Sogutlu G, Ara C, Yilmaz S, Kirimlioglu V (2005). Abdominal actinomycosis simulating malignancy of the right colon. Dig Dis Sci.

[3] Lee YM, Law WL, Chu KW (2001). Abdominal actinomycosis. Aust N Z J Surg.

[4] Wang YH, Tsai HC, Lee SS, Mai MH, Wann SR, Chen YS (2007). Clinical manifestations of actinomycosis in Southern Taiwan. J Microbiol Immunol Infect.

[5] Oksüz M, Sandikçi S, Culhaci A, Egesel T, Tuncer I (2007). Primary gastric actinomycosis: a case report. Turk J Gastroenterol.

[6] Lee SH, Kim HJ, Kim HJ, Chung IK, Kim HS, Park SH (2004). Primary gastric actinomycosis diagnosed by endoscopic biopsy: case report. Gastrointest Endosc.

[7] Evans J, Chan C, Gluch L, Fielding I, Eckstein R (1999). Inflammatory pseudotumour secondary to actinomyces infection. Aust N Z J Surg.

[8] Minamino H, Machida H, Tominaga K, Kameda N, Okazaki H, Tanigawa T (2011). A case report on primary gastric actinomycosis. Gastroenterol Endosc.

[9] Skoutelis A, Panagopoulos C, Kalfarentzos F, Bassaris H (1995). Intramural gastric actinomycosis. South Med J.

[10] Lee CM, Ng SH, Wan YL, Tsai CH (1996). Gastric actinomycosis. J Formos Med Assoc.

[11] Fernández-Aceñero MJ, Silvestre V, Fernández-Roldán R, Cortes L, Garcia-Blanch G (2004). Gastric actinomycosis: a rare complication after gastric bypass for morbid obesity. Obes Surg.

[12] Van Olmen G, Larmuseau MF, Geboes K, Rutgeerts P, Penninckx F, Vantrappen G (1984). Primary gastric actinomycosis: a case report and review of the literature. Am J Gastroenterol.

[13] Mazuji MK, Henry JS (1967). Gastric actinomycosis: case report. Arch Surg.

[14] Urdaneta LF, Belin RP, Cueto J, Doberneck RC (1967). Intramural gastric actinomycosis. Surgery.

[15] Figueras Felip J, Martín Rague J, Madesvall N, Norquera C, Casias AL (1979). Intramural gastric abscess. Rev Esp Enfer Apar Dig.

[16] Dellagi K, Kchir N, Mezni F, Boubaker S, el Quertani L, Zitouni MM (1986). Abdominal actinomycosis: a rare complication of gastric surgery? A propos of a case. Ann Gastroenterol Hepatol.

[17] Eastridge CE, Prather JR, Hughes FA (1972). Actinomycosis: a 24-year experience. South Med J.

[18] Wilson E (1961). Abdominal actinomycosis with special reference to the stomach. Br J Surg.

[19] Lee DL, Kang JY, Kim H, Lee KH, Choi GY, Jeon WJ (2009). A case of primary gastric actinomycosis. Korean J Med.

[20] Euanorasetr C, Sornmayura P (2010). Gastric Outlet Obstruction Secondary to Gastric Actinomycosis: A Case Report and Literature Review. The THAI Journal of SURGERY.

[21] Kaszuba M, Tomaszewska R, Pityñski K, Grzanka P, Bazan-Socha S, Musail J (2008). Actinomycosis mimicking advanced cancer. Pol Arch Med Wewn.

[22] Berardi RS (1979). Abdominal actinomycosis. Surg Gynecol Obstet.

[23] Sumer Y, Yilmaz B, Emre B, Ugur C (2004). Abdominal mass secondary to actinomyces infection: an unusual presentation and its treatment. J Postgrad Med.

[24] Huang CJ, Huang TJ, Hsieh JS (2004). Pseudo-colonic carcinoma caused by abdominal actinomycosis: report of two cases. Int J Colorectal Dis.

[25] Wagenlehner FM, Mohren B, Naber KG, Mannl HF (2003). Abdominal actinomycosis. Clin Microbiol Infect.

[26] Alam MK, Khayat FA, Al-Kayali A, Al-Suhaibani YA (2001). Abdominal actinomycosis: case reports. Saudi J Gastroenterol.

[27] Russo TA, Mandell GL, Bennett JE, Dolin R (1995). Agents of actinomycosis. Principles and practice of infectious diseases.

[28] Weese WC, Smith IM (1975). A study of 57 cases of actinomycosis over a 36-year period. Arch Intern Med.

[29] Yang SH, Li AF, Lin JK (2000). Colonoscopy in abdominal actinomycosis. Gastrointest Endosc.

[30] Brown JR (1973). Human actinomycosis: a study of 181 subjects. Hum Pathol.

[31] Srivastava A, Lauwers GY (2007). Pathology of non-infective gastritis. Histopathology.

[32] Das N, Lee J, Madden M, Elliot CS, Bateson P, Gilliland R (2006). A rare case of abdominal actinomycosis presenting as an inflammatory pseudotumor. Int J Colorectal Dis.

[33] Chattopadhyay P, Chatterjee S, Sen SK (2008). Biotechnological potential of natural food grade biocolorants. Afr J Biotech.

[34] Tunkel AR, Sepkowitz KA (2002). Infections Caused by Viridans Streptococci in Patients with Neutropenia. Clin Infect Dis.

[35] Brook I, Hunter V, Walker RI (1984). Synergistic effects of anaerobic ccocci, Bacteroides, Clostridia, Fusobacteria, and anaerobic bacteria on mouse and induction of substances abscess. J Infect Dis.

